# Whole blood microRNA expression pattern differentiates patients with rheumatoid arthritis, their seropositive first-degree relatives, and healthy unrelated control subjects

**DOI:** 10.1186/s13075-017-1459-x

**Published:** 2017-11-10

**Authors:** Vidyanand Anaparti, Irene Smolik, Xiaobo Meng, Victor Spicer, Neeloffer Mookherjee, Hani El-Gabalawy

**Affiliations:** 1 Department of Internal Medicine, Rady Faculty of Health Sciences, University of Manitoba, Room 799, 715 McDermot Avenue, Winnipeg, MB R3E 3P4 Canada; 2 Manitoba Centre for Proteomics and Systems Biology, University of Manitoba, Winnipeg, MB Canada; 3 Rheumatic Diseases Unit, University of Manitoba, Winnipeg, MB Canada; 4 Division of Rheumatology, Faculty of Health Sciences, University of Manitoba, Winnipeg, MB Canada; 5 Department of Immunology, Rady Faculty of Health Sciences, University of Manitoba, Winnipeg, MB Canada

**Keywords:** MicroRNA, Rheumatoid arthritis, Epigenetics, miRNA, miR-103a-3p, Whole blood, miR-103a

## Abstract

**Background:**

Epigenetic mechanisms can integrate gene-environment interactions that mediate disease transition from preclinical to clinically overt rheumatoid arthritis (RA). To better understand their role, we evaluated microRNA (miRNA, miR) expression profile in indigenous North American patients with RA who were positive for anticitrullinated protein antibodies; their autoantibody-positive, asymptomatic first-degree relatives (FDRs); and disease-free healthy control subjects (HCs).

**Methods:**

Total RNA was isolated from whole blood samples obtained from HC (*n* = 12), patients with RA (*n* = 18), and FDRs (*n* = 12). Expression of 35 selected relevant miRNAs, as well as associated downstream messenger RNA (mRNA) targets of miR-103a-3p, was determined by qRT-PCR.

**Results:**

Whole blood expression profiling identified significantly differential miRNA expression in patients with RA (13 miRNAs) and FDRs (10 miRNAs) compared with HCs. Among these, expression of miR-103a-3p, miR-155, miR-146a-5p, and miR-26b-3p was significantly upregulated, whereas miR-346 was significantly downregulated, in both study groups. Expression of miR-103a-3p was consistently elevated in FDRs at two time points 1 year apart. We also confirmed increased miR-103a-3p expression in peripheral blood mononuclear cells from patients with RA compared with HCs. Predicted target analyses of differentially expressed miRNAs in patients with RA and FDRs showed overlapping biological networks. Consistent with these curated networks, mRNA expression of *DICER1*, *AGO1*, *CREB1*, *DAPK1*, and *TP53* was downregulated significantly with miR-103a-3p expression in FDRs.

**Conclusions:**

We highlight systematically altered circulating miRNA expression in at-risk FDRs prior to RA onset, a profile they shared with patients with RA. Prominently consistent miR-103a-3p expression indicates its utility as a prognostic biomarker for preclinical RA while highlighting biological pathways important for transition to clinically detectable disease.

**Electronic supplementary material:**

The online version of this article (doi:10.1186/s13075-017-1459-x) contains supplementary material, which is available to authorized users.

## Background

Rheumatoid arthritis (RA) is a chronic autoimmune disease that results from a complex interplay between genetics, environmental factors, and the immune system. Retrospective studies of RA onset based on archival serum samples have indicated that rheumatoid factor (RF) and anticitrullinated protein antibodies (ACPA) are detectable months to years prior to clinical disease onset, and they exhibit a progressive increase in titer as disease onset approaches. In the case of ACPA, this phenomenon is believed to relate to expansion of an autoantigen repertoire targeted by the ACPA, a process that has been termed *epitope spreading* [1–3].

We previously demonstrated a high prevalence of RA in an indigenous North American (INA) population in Manitoba, Canada, an observation that is consistent with those in other INA populations [4]. In this population, RA is characterized by familial disease aggregation and early age of disease onset [5, 6]. A high proportion of these INA patients with RA are genetically predisposed by having shared epitope encoding HLA-DRB1 alleles, particularly *1402 and *0404 [7]. The disease is primarily seropositive, and it is severe and disabling, with frequent large joint involvement. In studying the first-degree relatives (FDRs) of INA patients with RA, we have demonstrated frequent RF and ACPA seropositivity, and we have shown that the serum cytokine profile of the FDRs resembles that of their affected relatives more so than that of control subjects with no family history of autoimmune disease [6–8]. Thus, this population is ideally suited for studying the onset of RA in high-risk individuals and the potential role that genetic, environmental, and epigenetic factors play in the process.

MicroRNAs (miRNAs, miRs) are conserved, small, noncoding, single-stranded RNAs (~18–25 nucleotides) that play a role in posttranscriptional gene regulation. miRNAs bind to the 3′-untranslated region (3′-UTR) of target messenger RNA (mRNA) and induce gene silencing by either promoting mRNA degradation or transcript destabilization, resulting in suppression of target protein synthesis [9, 10]. In RA, proinflammatory cytokines (e.g., tumor necrosis factor-α, interleukin [IL]-1β, and IL-17) alter the expression of multiple miRNAs (e.g., miR-155, miR-146a, miR-26b, miR-16, and miR-21) in peripheral blood mononuclear cells (PBMCs), synovial fibroblasts, T lymphocytes, and synovial tissues derived from patients with RA [11–13]. In turn, miRNAs regulate inflammatory and signaling pathways influencing cellular differentiation and bone homeostasis within the synovial microenvironment [11]. Consequently, miRNAs play a central role in the regulation of inflammatory processes, synovial proliferation, and osteoclastogenesis, thus affecting the disease activity in RA [12–14]. Therefore, miRNAs may serve as a critical epigenetic component in the breakdown of immune tolerance and progression toward RA disease onset.

There is limited knowledge on the role of miRNAs in RA pathogenesis, particularly during the preclinical phase of the disease. To define mechanisms underpinning the progression of autoimmunity toward disease onset in at-risk individuals, we sought to evaluate miRNA expression profiles in blood samples derived from INA patients with RA, their seropositive FDRs, and healthy control subjects (HCs). This is the first study to demonstrate unique and reproducible differences in miRNA expression patterns in whole blood between these groups. Furthermore, we demonstrated that miR-103a-3p is uniquely upregulated in both patients with RA and FDRs. The observed miRNA patterns and the molecular networks they represent are of value in defining new mechanisms involved in RA onset while being potentially useful as biomarkers for predicting onset of preclinical RA.

## Methods

### Study design

INA study participants were recruited from Cree, Ojibway, and Oji-Cree communities in central Canada [5, 6]. The biomedical research ethics board of the University of Manitoba approved the overall design of the study and the consent forms (ethics, 2005:093; protocol, HS14453). Specific research agreements with the study communities were developed and approved by the community leadership. The conduct of the study was guided by the principles of community-based participatory research, a cornerstone of the Canadian Institutes of Health Research guidelines for Aboriginal health research (http://www.cihr-irsc.gc.ca/e/29134.html). As such, community leadership provided input into the initial development of the project, as well as ongoing input through advisory board meetings. Local healthcare providers were trained in study methodology and standard operating procedures. Regular knowledge translation activities such as newsletters and local radio appearances by study investigators provided the communities with updates regarding progress and significance. The study participants provided informed consent after the study was explained to them in detail, with the help of an INA translator from their community where necessary. The following three groups were included in this study: (1) ACPA-positive patients with RA, (2) their unaffected ACPA-positive FDRs, and (3) HCs negative for ACPA and RF. The demographics of the study groups are summarized in Table 1. RA diagnosis was made on the basis of fulfilling the 2010 American College of Rheumatology/European League Against Rheumatism classification criteria. None of the FDRs or HCs demonstrated clinical evidence of synovitis, as determined by a rheumatologist (HEG).

**Table 1:** Clinical characteristics of the study population

	Healthy control subjects (*n* = 12)	ACPA+/patients with RA (*n* = 18)	ACPA+/FDRs (*n* = 12)
Age, years, median (range)	40 (23–66)	46.6 (29–70)	33.65 (28–60)
Sex, female/male	10/2	14/4	12/1
Disease duration, years, median (range)	NA	12.02 (0–35.6)	NA
CRP titer, median (range)	3.35 (1.07 –9.25)	6.91 (2–42.6)	2.595 (1.01–15.9)
RF titer, IU/ml, median (range)ml	<20	321 (20–1540)	34.9 (20–570)
Anti-CCP titer, median (range)	1 (0.4–2.0)	201 (19–289)	114 (7–365)
BMI, kg/m^2^, median (range)	29.54 (19.9–34.4)	27.37 (20.4–39.6)	26.09 (19.6–40.7)

*Abbreviations: ACPA* Anticitrullinated protein antibodies, *BMI* Body mass index, *Anti-CCP* Anticyclic citrullinated protein antibodies, *CRP* C-reactive protein, *FDR* First-degree relative, *RA* Rheumatoid arthritis, *RF* Rheumatoid factor, *NA* Not applicable

All values are reported as median (range) unless otherwise indicated

### Sample collection

Venous blood was collected into PAXgene® Blood RNA tubes (PreAnalytiX, Hombrechtikon, Switzerland), processed as per the manufacturer’s instructions, and used to isolate total RNA. PBMCs were isolated using SepMate®-50 tubes (STEMCELL Technologies, Vancouver, BC, Canada) as per the manufacturer’s protocol. Briefly, venous blood was drawn into ethylenediaminetetraacetic acid-coated tubes and diluted 1:1 with incomplete Gibco RPMI medium (Life Technologies, Carlsbad, CA, USA), layered onto SepMate®-50 tubes with Histopaque Plus (Sigma-Aldrich, St. Louis, MO, USA), and centrifuged at 1000 × *g* for 10 minutes at room temperature. Buffy coat was separated, and cells were washed in RPMI 1640 medium prior to RNA isolation.

### Immunoassays

Serum C-reactive protein (CRP) levels were monitored in serum by using a human high-sensitivity C-reactive protein (hs-CRP) enzyme-linked immunosorbent assay kit (Biomatik, Cambridge, ON, Canada) as per the manufacturer’s instructions. The concentration of ACPA was monitored in serum using the BioPlex® 2200 anticyclic citrullinated protein antibodies reagent kit (Bio-Rad Laboratories, Hercules, CA, USA).

### Total RNA extraction and qRT-PCR

Total RNA was isolated from whole blood and PBMCs using the Ambion *mir*VANA miRNA isolation kit (catalogue number AM1561; Life Technologies, Carlsbad, CA, USA) as per the manufacturer’s instructions. RNA quality was determined using Bioanalyzer with the RNA 6000 Nano Kit (Agilent Technologies, Santa Clara, CA, USA). Total RNA with absorbance at 260 and 280 nm ≥ 2.0 and RNA integrity number ≥ 7.0 was used for monitoring miRNA expression using a two-step qRT-PCR protocol as previously described [15]. Briefly, we used the Applied Biosystems TaqMan® MicroRNA Reverse Transcription Kit (Life Technologies) with miRNA-specific stem-loop primers for reverse transcription (Additional file 1: Table S1). Specific amplification of miRNA targets was performed using TaqMan® Universal Master Mix II and target-specific TaqMan® MicroRNA Assay Mix in the ABI PRISM 7300 Real-Time PCR System (all from Life Technologies). For mRNA amplification, first-strand complementary DNA was synthesized from total RNA (1 μg) using SuperScript® VILO^TM^ MasterMix (Life Technologies) as per the manufacturer’s instructions. Target mRNA was amplified using Applied Biosystems® *Power* SYBR® Green Master Mix (Life Technologies) as per the manufacturer’s instructions. Primers used for mRNA amplification of miR-103a-3p are listed in Additional file 1: Table S2.

### Data analysis and statistics

Candidate endogenous control miRNAs for data normalization were selected on the basis of prior literature (RNU48, RNU44, U6 snRNA, RNU6B, and miR-16). Expression of these selected miRNAs was assessed for stable expression across samples in whole blood and PBMCs obtained from HCs, patients with RA, and FDRs. RefFinder, a web-based comprehensive gene analysis platform that integrates geNorm, NormFinder, BestKeeper, and comparative cycle threshold (Δ*C*_t_) methods, was used to identify the miRNA candidates suitable as endogenous controls for data normalization. On the basis of this approach, RNU48 and RNU6B were identified as optimum reference miRNAs for normalization across all samples in this study [16]. Reference *C*_t_ values for data normalization were determined by calculating the average *C*_t_ value of RNU48 and RNU6B [reference *C*_t_ = mean (*C*_t_ {RNU48} − *C*_t_ (RNU6B)] and used for each sample. Raw *C*_t_ values for each target miRNA were then normalized with reference *C*_t_ values to obtain Δ*C*_t_ values for each sample [Δ*C*_t_ (target miRNA) = *C*_t_ (target) − reference *C*_t_]. Δ*C*_t_ values of each miRNA were further corrected using a global mean normalization strategy to obtain normalized Δ*C*_t_ values [normalized Δ*C*_t_ = Δ*C*_t_ (target miRNA) − mean Δ*C*_t_] for all assessed miRNAs [17–19]. Relative fold changes were calculated using the ΔΔ*C*_t_ method [20]. Of the 35 miRNAs analyzed, 33 showed detectable expression (*C*_t_ ≤ 35) (Table 2) and were considered for further analyses. Target mRNA expression was determined in samples after normalization using 18S ribosomal RNA as an endogenous control [20], and relative fold changes were calculated using the ΔΔ*C*_t_ method.

**Table 2:** Fold change expression of microRNAs

miRNA	RA vs HC			FDR vs HC		FDR vs RA	
	*P* Value	Fold change	Rank	*P* Value	Fold change	*P* Value	Fold change
**hsa-miR-103a-3p**	**0.0064**	**3.96**	**1**	**0.0238**	**7.68**	0.1223	1.97
**hsa-miR-155**	**0.0002**	**2.47**	**2**	**0.0115**	**1.98**	0.3627	−1.25
hsa-miR-29b	0.0648	1.91	3	0.8636	−1.55	0.0754	−2.96
**hsa-miR-132**	**0.0016**	**1.90**	**4**	0.0687	1.37	0.2530	−1.39
**hsa-miR-26b-3p**	**0.0010**	**1.88**	**5**	**0.0024**	**2.28**	0.2530	1.21
**hsa-miR-152**	**0.0038**	**1.83**	**6**	0.1309	1.97	0.4981	1.08
**hsa-miR-19a**	0.0732	1.73	**7**	**0.0205**	**1.55**	0.9662	−1.11
hsa-Let-7a	0.0569	1.73	**7**	0.2086	1.17	0.2358	−1.47
**hsa-miR-19b**	**0.0260**	**1.67**	**9**	0.1169	1.34	0.4091	−1.24
**hsa-miR-146a-5p**	**0.0083**	**1.54**	**10**	**0.0031**	**1.99**	0.3408	1.29
**hsa-miR-451**	**0.0076**	**1.53**	**11**	**0.0127**	**1.56**	0.7031	1.02
**RNU44**	**0.0120**	**1.52**	**12**	**0.0162**	**1.30**	0.9157	−1.17
**hsa-miR-125a-5p**	**0.0272**	**1.30**	**13**	0.0553	1.50	0.7508	1.15
hsa-miR-222	0.0796	1.29	14	0.2154	1.20	0.4587	−1.07
hsa-miR-107	0.0576	1.28	15	0.2481	−1.02	0.4587	−1.31
hsa-miR-29c	0.2428	1.28	16	0.1457	1.11	0.8159	−1.15
hsa-Let-7e	0.0502	1.27	17	0.5837	−1.20	0.0987	−1.53
**hsa-miR-21**	**0.0261**	**1.24**	**18**	0.0924	1.22	0.7832	−1.16
**hsa-miR-223**	**0.0115**	**1.22**	**19**	0.5487	1.03	0.5115	−1.82
**hsa-miR-26b-5p**	0.1191	1.21	20	0.6744	−1.45	**0.0277**	**−1.76**
hsa-miR-323-3p	0.1123	1.17	21	0.2033	1.12	0.9831	−1.04
hsa-miR-26a	0.1849	1.14	22	0.1159	1.33	0.8822	1.16
hsa-miR-29a	0.1031	1.14	23	0.6174	−1.29	0.1124	1.46
hsa-miR-15a	0.5594	1.11	24	0.8292	−1.56	0.1223	−1.72
hsa-miR-150	0.6104	1.10	25	0.41	1.20	0.7669	1.09
hsa-miR-34a*	0.1065	1.06	26	**0.0495**	**−1.84**	**0.0262**	**−1.94**
hsa-miR-221	0.1842	1.06	27	0.9914	−1.37	0.2356	−1.45
hsa-miR-24	0.2956	−1.05	28	0.8651	1.03	0.9831	1.04
hsa-miR-18a	0.7615	−1.07	29	0.7639	−1.85	0.1124	−1.72
**U6**	0.8069	−1.11	30	**0.0001**	**−1.59**	0.0625	−1.43
hsa-miR-125a-3p	0.1654	−1.20	31	0.6178	−1.10	0.4847	1.10
hsa-miR-16	0.9950	−3.94	32	0.8276	−1.24	0.2802	−1.23
**hsa-miR-346**	**0.0001**	**−8.70**	**33**	**0.0001**	**−20.00**	**0.0338**	**−2.32**

*Abbreviations: FDR* First-degree relative, *HC* Healthy control subjects, *miRNA* or *miR* MicroRNA, *RA* Rheumatoid arthritis

miRNA profiling was analyzed in the whole blood total RNA samples obtained from HC, patients with RA, and FDRs using TaqMan probes. *P* values were obtained using the Mann-Whitney *U* test, and median fold change values were used to classify miRNAs rankwise. Columns highlighted in *bold* represent miRNAs that show significant differential expression after Benjamini-Hochberg correction for multiple comparison analyses (*Q* < 0.04). RA vs HC indicates miRNA expression in patients with RA compared with HCs. FDR vs HC indicates miRNA expression in FDRs compared with HCs. FDR vs RA indicates miRNA expression in FDRs compared with patients with RA. hsa-miR-34a* represents hsa-miR-34a-3p

GraphPad Prism version 5.0 was used for miRNA analysis and generating volcano plots, scatterplots, and bar graphs. Empirical cumulative distribution plots (based on the Kolmogorov-Smirnov [KS] test) and ROC curves were generated using MS Excel (Microsoft, Redmond, WA, USA) and Prism (GraphPad Prism, La Jolla, CA, USA) software, respectively. The KS test is a nonparametric statistical method that does not assume normal distribution [21]. Differences between the datasets were represented as KS scores (in the range of −1 and 1) corresponding to maximum degree of separation between the cumulative distributions of the datasets being compared and directly proportional to relative expression levels. KS scores > 0.5 were considered significant. Heat maps were generated with unsupervised hierarchical clustering using the TIGR multiple experiment viewer. Ingenuity Pathway Analysis ([IPA] www.ingenuity.com; QIAGEN Bioinformatics, Redwood City, CA, USA) was used for biomolecular network analyses and to predict mRNAs targeted by the differentially expressed miRNAs identified in this study. The Mann-Whitney *U* test, the Kruskal-Wallis test with Dunn’s post hoc method, or Spearman’s rank correlation coefficient analysis was used for statistical analysis as required, and *P* values < 0.05 were considered significant. Differentially expressed miRNAs were determined after adjusting *P* values with Benjamini-Hochberg correction for multiple comparisons [22].

## Results

### Study population

Participants were age-matched, ethnically homogeneous individuals, and approximately 80% of them were women (Table 1). As expected, patients with RA demonstrated higher hs-CRP levels (mean ± SD 10.05 ± 10.02 μg/ml) than HCs (mean ± SD 4.03 ± 2.31 μg/ml) and FDR (mean ± SD 4.18 ± 3.09 μg/ml). Patients with RA in the study were on disease-modifying antirheumatic drugs and had an established disease profile that was either inactive or moderately active, as indicated by their Disease Activity Score in 28 joints (Table 1 and Additional file 1: Table S3).

### Whole blood miRNA expression profile was altered in patients with RA and FDRs

**Fig. 1:**
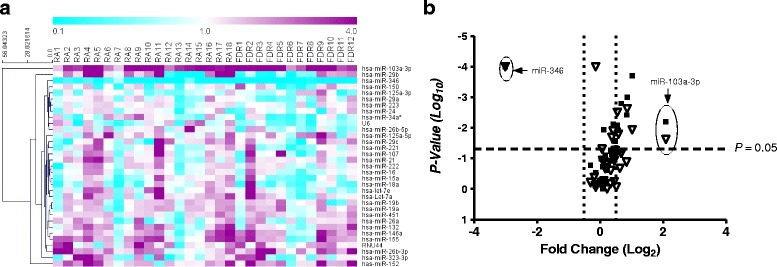
**a** Unsupervised hierarchical clustering generated using fold change expression values of microRNAs (miRNAs, miRs) analyzed in patients with rheumatoid arthritis (RA) and first-degree relatives (FDRs) compared with healthy control subjects. Color scheme: *Violet* = increased expression; *cyan* = decreased expression; and *white* = unchanged. **b** Volcano plot showing expression of 33 miRNAs in the whole blood of patients with RA (*closed squares*) and FDRs (*open triangles*). The miRNAs that are significantly altered in both groups are located above the *horizontal dashed line* corresponding to *P* ≤ 0.05

Using targeted TaqMan® miRNA assay probes (Life Technologies), we analyzed the expression of 33 selected miRNAs. The miRNAs were selected on the basis of their relevance to RA as described in the literature (Additional file 1: Table S1). Overall, our analysis indicated that RA and FDR groups exhibited uniquely similar miRNA expression patterns compared with HC in whole blood samples (Table 2 and Fig. 1; Additional file 1: Table S4), but there were notable differences between these three groups. Whereas expression of 13 miRNAs was significantly different in patients with RA, 10 miRNAs were differentially expressed in FDRs, compared with HCs. Notably, the expression of miR-103a-3p was increased in both patients with RA (~3.96-fold) and FDR (~7.68-fold), whereas the expression of miR-346 was decreased in both groups (~8.7-fold and ~ 20-fold, respectively). Finally, in comparing patients with RA with FDRs, miR-34a*, miR-26b-5p, and miR-346 differed significantly in their expression levels.

Unsupervised hierarchical clustering (Fig. 1a) of all 33 detectable miRNAs in patients with RA and FDRs was performed to generate a tree clearly separating miRNAs into two major clusters. Volcano scatter plots further demonstrated that miR-103a-3p and miR-346 were the most upregulated (upper right corner of the plot) and downregulated (upper left corner of the plot), respectively (Fig. 1b). These findings suggested that miR-103a-3p was uniquely upregulated in both patients with RA and their FDRs compared with HCs. On this basis, we undertook further analyses to examine the performance of miR-103a-3p as a biomarker in this population.

### Performance of miR-103a-3p as a biomarker

**Fig. 2:**
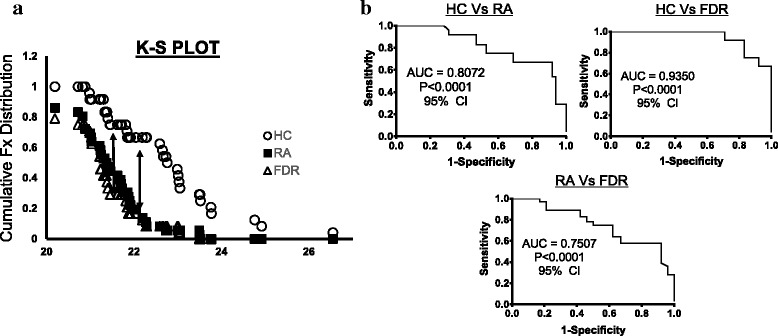
**a** Kolmogorov-Smirnov (K-S) plots showing cumulative distribution of microRNA (miR) miR-103a-3p expression for healthy control subjects (HCs; *open circles*), patients with rheumatoid arthritis (RA; *closed squares*), and first-degree relatives (FDRs; *open triangles*). The distance between two distribution plots is represented by *double arrows*. K-S distance ≥ 0.5 was considered significant. **b** ROC curves (representing 1-specificity vs sensitivity values) for HCs, patients with RA, and FDRs, calculated using expression values of miR-103a-3p

Empirical cumulative distribution plots, together with ROC analysis, showed that miR-103a-3p can effectively distinguish between HCs, patients with RA, and FDRs (*P* < 0.0001; 0.01% false discovery rate). The calculated KS distance between HCs and patients with RA based on miR-103a-3p expression was 0.59 (at Δ*C*_t_ = 22.29), whereas FDRs were separated by 0.75 (at Δ*C*_t_ = 21.32) from HCs and by 0.49 (at Δ*C*_t_ = 20.89) from patients with RA (Fig. 2a). We determined the sensitivity and specificity of miR-103a-3p expression using KS distance as a cutoff point (Fig. 2b). At 95% CI, the AUC of the ROC plot was 0. 8072 for ACPA-positive patients with RA (*P* < 0.0001; 92% specificity and 67% sensitivity), whereas FDRs showed AUCs of 0.9350 (*P* < 0.0001; 92% specificity and 83% sensitivity) compared with HCs and 0.7507 (*P* < 0.001; 71% specificity and 78% sensitivity) compared with patients with RA. These analyses suggest that elevated whole blood levels of miR-103a-3p may serve as a robust biomarker in ACPA-positive individuals at risk for developing future RA.

### Elevated whole blood miR-103a-3p expression levels are stable feature in FDRs

**Fig. 3:**
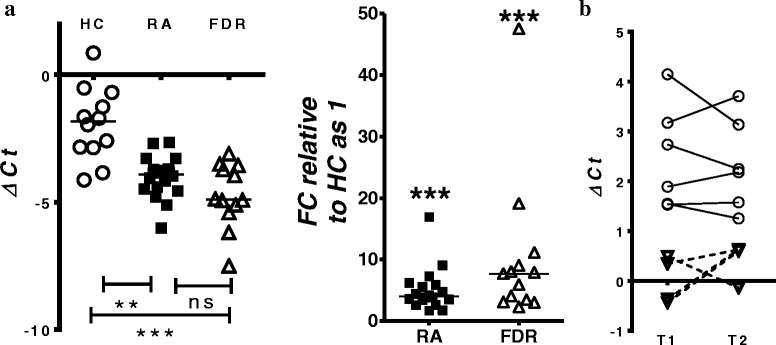
Expression of microRNA (miR) miR-103a-3p in whole blood or peripheral blood mononuclear cells obtained from healthy control subjects (HCs), patients with rheumatoid arthritis (RA), or first-degree relatives (FDRs). miR-103a-3p transcriptional abundance was analyzed by qPCR. **a** Scatterplot of normalized cycle threshold (Δ*C*_t_) values and fold change of miR-103a-3p relative expression in whole blood of HCs (*open circles*), patients with RA (*filled squares*), and FDRs (*open triangles*). Error bars represent median values analyzed by Kruskal-Wallis test with Dunn’s test for multiple comparisons (*** *P* < 0.001 vs HCs). **b** Scatterplot of normalized Δ*C*_t_ values showing miR-103a-3p expression in whole blood of HCs (*open circles*) and FDRs (*open triangles*) collected at two independent time points ~ 1 year apart (T1 and T2)

FDRs, as a group, showed a ~ 7.6-fold increase in miR-103a-3p expression compared with HCs and a ~ 1.96-fold increase compared with patients with RA (Fig. 3a). We then sought to determine whether the increased miR-103a-3p expression levels were stable over time in specific individuals. Whole blood was collected from six HCs and two FDRs at two independent time points (~1 year apart), and miR-103a-3p expression was compared at the two time points. These experiments demonstrated that at both time points, the expression of miR-103a-3p was higher in FDRs (as indicated by lower Δ*C*_t_ values) compared with HCs (Fig. 3b). This suggests that there is sustained upregulation of miR-103a-3p in FDRs compared with HCs. We observed limited variability in miR-103a-3p expression related to time between sampling, sample acquisition, and sample storage (data not shown). The delineation of the relative contributions of various cellular subsets in whole blood to the observed increase in miR-103a-3p requires further experiments where individual cellular subsets are fractioned and tested.

### Pairwise miR-103a-3p target correlation analysis distinctly segregates FDRs

Biomolecular interaction between the differentially expressed miRNAs (Table 2) with their respective annotated transcript targets was analyzed using the IPA bioinformatics tool. The biomolecular network revealed that tumor protein 53 (TP53) and Argonaute 2 (AGO2) were the two major hubs within the network that were proposed to regulate the expression of most of the differentially expressed miRNAs identified in this study (Additional file 1: Figure S1). The curated functional pathways (log *P* value > 2.0 by Fisher’s exact test; threshold value = 0.05) predicted to be regulated by the miRNAs identified in this study included metabolic and physiological processes such as cellular growth, development, proliferation, and cell death and are overrepresented in chronic diseases (Additional file 1: Table S5).

**Fig. 4:**
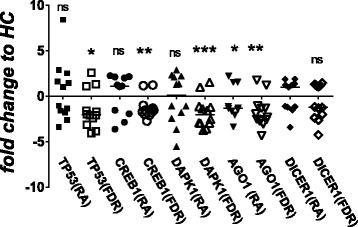
Transcript abundance of microRNA (miR) miR-103a-3p target messenger RNAs (DICER1, AGO1, CREB1, TP53, and DAPK1) analyzed by qPCR using total RNA obtained from whole blood of healthy control subjects (HCs), patients with rheumatoid arthritis (RA) and first-degree relatives (FDRs). Scatterplots represent relative fold change expression values compared with HCs. Error bars represent median values analyzed by Mann-Whitney *U* test. ***P < 0.001, ** *P* < 0.01, *P < 0.05. *ns* Nonsignificant compared with HCs

Consistent with curated network analysis, available literature suggests that miR-103a-3p expression is regulated by *TP53* and *AGO2* [23–29]. Furthermore, miR-103a-3p binds to 3′-UTRs of *CCNE*, *CDK1*, *DICER1*, *AGO1*, *GPD1*, *ID2*, *CREB1*, *TIMP3*, *DAPK1*, *KLF4*, and *PTEN* and regulates diverse physiological functions, including vascular inflammation, glucose metabolism, adipogenesis, endothelial cell activation, tumor metastasis, cellular apoptosis, and oxidative stress. Therefore, we monitored the expression of all the above-mentioned mRNAs by qRT-PCR (Fig. 4 and Additional file 1: Table S6). Compared with HCs, whole blood expression of Argonaute 1 (AGO1), cyclic AMP-responsive element-binding protein 1 (CREB1), death-associated protein kinase 1 (DAPK1), and TP53 was significantly downregulated in FDRs. DICER1 mRNA expression showed a similar trend, albeit statistically nonsignificant. No significant change was observed in whole blood of patients with RA compared with HCs, except for AGO1. Additionally, Spearman’s correlation analysis with Benjamini-Hochberg correction for multiple comparisons (Additional file 1: Table S6) did not demonstrate any statistical significance between miR-103a-3p and any of its target mRNA expression levels. This suggests that regulation of miR-103a-3p and its targets is complex and warrants further investigation.

## Discussion

In the present study, we examined the expression pattern of a wide spectrum of miRNAs in whole blood samples from a cohort of INA patients with RA, their ACPA-positive unaffected FDRs, and unaffected INA control subjects with no clinical or serological evidence of autoimmunity. We demonstrated distinct differences between all three groups, and to our knowledge, we are the first to demonstrate that miR-103a-3p is overexpressed in patients with RA and FDRs compared with HCs. Although aberrant miRNA expression patterns in the peripheral blood of patients with RA has been widely reported [13, 30], aberrant expression in ACPA-positive unaffected individuals has not been reported previously. This study provides an impetus for evaluating the whole blood miRNA profile, particularly miR-103a-3p expression, as a potential biomarker for predicting imminent disease in individuals at risk for developing RA. It also points to specific biological pathways that may be involved in the transition to clinically detectable disease.

We elected to examine miRNA profiles using whole blood samples collected in PAXgene® RNA tubes for several reasons. First and foremost is the ease with which these samples are collected and stored, along with the remarkable resistance of the miRNA to endogenous ribonuclease activity, as well as stability to extreme pH, temperature, and storage conditions [31, 32]. An alternative approach that is being widely investigated in a spectrum of chronic diseases is testing miRNA levels in serum or plasma [33, 34]. Although this latter cell-free approach has the advantage of potentially harnessing large archival serum/plasma sample repositories, it suffers from limitations in providing a complete and unbiased miRNA profile of the circulating peripheral blood compartment of an individual. This relates to factors such as preprocessing of samples, cellular contamination, and inconsistency in miRNA levels in serum vs plasma [35, 36].

One major advantage of using whole blood to determine miRNA levels is that this approach retains the rich compositional architecture of the circulating blood, thus providing the most unbiased representation of this space. Although this approach may be ideally suited for biomarker discovery, its primary disadvantage is the inability to define the cellular subsets that are contributing to the observed miRNA profiles. Combined with the marked cellular compositional heterogeneity of whole blood, the generation of mechanistic hypotheses is challenging. To address this challenge, most of the previous studies of circulating miRNA expression in RA have been focused on PBMCs and their subsets [13]. However, attempts to correlate PBMC expression patterns with those evident in whole blood have produced conflicting results [15]. For instance, Atarod et al. demonstrated discordant expression of miR-146a-5p and miR-155 expression between PBMCs and whole blood [37]. These findings contradict the findings of our previous study [15], which demonstrated more concordance between whole blood and PBMC expression patterns. These differences may be attributable to total RNA isolation methodology used in each of these sample types. Alternatively, this discordance can also be attributed to blood cell counts and red blood cell hemolysis [38]. We acknowledge the absence of such information pertaining to our study participants.

Previous studies on miRNA expression in RA, including our own, have been focused on differences in miR-146a and miR-155 expression between patients with RA and unaffected control subjects, both tending to be increased in RA PBMCs and synovial tissues [13, 15, 39]. In the present study, we compared the expression levels of these two miRNAs in whole blood and PBMCs and found that the levels were concordantly elevated not only in patients with RA as previously documented but also, surprisingly, in ACPA-positive FDRs with no clinical evidence of arthritis. Moreover, as shown in Fig. 1, the overall miRNA expression patterns in patients with RA and ACPA-positive FDRs were relatively similar to those of unaffected control subjects. These observations suggest that the similarity between patients with RA and unaffected ACPA-positive FDRs in the peripheral blood miRNA profile is more likely to relate to autoimmune than to inflammatory mechanisms. Moreover, we demonstrated that these patterns are relatively stable over a short time frame. It will be of interest to determine how the miRNA patterns evolve as individuals at risk for developing RA transition to clinically detectable synovitis. It will also be of interest to determine whether these RA-associated patterns are retained in patients with RA who have achieved clinical remission.

The large difference in miR-103a-3p expression that discriminated both patients with RA and FDRs from unaffected, population-based control subjects is noteworthy and, to our knowledge, not previously reported. Located within the intronic regions of pantothenate kinase enzymes, miR-103a-3p is a member of the miR-15/107 cluster and regulates lipid, cholesterol, and fatty acid metabolism; adipocyte differentiation; and insulin signaling [40–42]. However, the potential role that these biological functions play in RA pathogenesis remains largely unknown. Some studies have suggested that miR-103 upregulation is associated with obesity and insulin resistance in liver and adipose tissue, as well as with atherosclerosis [24, 43, 44]. Interestingly, the indigenous First Nations population as a whole, including the cohort we have studied, demonstrates a strikingly high prevalence of obesity, type 2 diabetes, and cardiovascular disease [45, 46].

To identify potential gene targets of miR-103a-3p and delineate the biological functions that they regulate, we performed computational predictive analysis using IPA. On the basis of the curated IPA target network analysis, we identified TP53 and AGO2 as central nodes in miRNA patterns detected in patients with RA and ACPA-positive unaffected FDRs. AGO2 is an integral component of RNA-induced silencing complex (RISC) that cleaves double-stranded immature miRNAs to single-stranded mature forms, a reaction catalyzed by an RNase III-type enzyme called Dicer [9]. Altered TP53 expression has been observed in lymphocytes and synovial tissues from patients with RA and is associated with synovial proliferation and increased proinflammatory IL-6 secretion in the synovium [47, 48]. Interestingly, miR-103a-3p associates with AGO2 within RISC and is known to suppress Dicer [24, 49]. TP53 also regulates miR-103 expression via targeting components of miRNA biogenesis, including DICER1 and AGO2 [50]. Together, our observations point to miR-103a-3p-associated miRNA target reorganization in patients with RA and ACPA-positive FDRs at risk for developing RA. It is notable that the regulatory networks of miRNAs, including miR-103a-3p and its target mRNAs, are extremely complex and known to control physiological processes at multiple levels [51]. Considering that miRNAs are involved in an intricate network of feedback and feedforward regulatory loops, it is likely that the target mRNAs monitored in our study may modulate the expression of other miRNAs [52, 53]. In this regard, further research is warranted to investigate the interaction network between miR-103a-3p and its target mRNAs in different cohorts, especially FDRs, to examine biological processes prior to onset of RA.

## Conclusions

We present evidence that the miRNA signature detectable in the peripheral blood of ACPA-positive individuals with no clinical evidence of RA resembles that of seropositive patients with RA and that this pattern differs considerably from that seen in unaffected seronegative controls. The substantial elevation of miR-103a-3p levels compared with unaffected control subjects is particularly discriminating and, in conjunction with phenomena such as the epitope spreading of the ACPA response, may serve as a potential biomarker for imminent RA in at-risk individuals. Longitudinal studies will be needed to determine how this miRNA signature evolves as individuals develop clinical disease and detectable synovitis. This in turn will provide new insights into the biological mechanisms underlying this important transition point.

## Additional file

Additional file 1: Table S1. Details of miRNAs included in the study. **Table S2.** Primers for mRNA targets of miR-103a-3p. Primers were designed using the PrimerQuest tool (Integrated DNA Technologies, Coralville, IA, USA) and the Universal ProbeLibrary system (Roche Life Sciences, Indianapolis, IN, USA) and verified by Primer-BLAST (National Center for Biotechnology Information, Bethesda, MD, USA). 18S ribosomal RNA, *F* Forward, *R* Reverse. **Table S3.** Clinical features of ACPA-positive patients with RA. **Table S4.** Differentially expressed miRNAs in probands and FDRs: significantly upregulated (↑) and downregulated (↓) or unaltered miRNAs. ^#^ Only miRNAs differentially expressed in FDRs compared with patients with RA. **Figure S1.** IPA target analysis. IPA network showing curated molecular interactions between differentially expressed miRNAs and their experimentally validated target genes. **Table S5.** Summary of findings derived from IPA regarding gene targets of differentially expressed miRNAs: top molecular and cellular functions, physiological system development and function, diseases and disorders, and networks regulated by significantly modulated miRNAs and their gene targets (*P* < 0.05 by Fisher’s exact test). **Table S6.** Spearman’s rank correlation coefficient values and corresponding *P* values for miR-103a-3p and target mRNAs. ΔC_t_ values of miR-103a-3p Vs target mRNAs from HC, patients with RA, and FDRs were combined for this correlation analysis. *Q* values represent adjusted *P* values after applying the Benjamini-Hochberg correction for multiple comparisons. (DOCX 836 kb)

## Abbreviations

ACPA: Anticitrullinated protein antibodies
AGO1: Argonaute 1
AGO2: Argonaute 2
Anti-CCP: Anticyclic citrullinated protein antibodies
CCNE1: Cyclin E1
CDK1: Cyclin-dependent kinase 1
CIHR: Canadian Institutes of Health Research
CREB1: Cyclic AMP-responsive element-binding protein 1
CRP: C-reactive protein
ΔC_t_: Cycle threshold method
DAPK1: Death-associated protein kinase 1
DICER1: Dicer 1, ribonuclease III
FDR: First-degree relative
GPD1: Glycerol-3-phosphate dehydrogenase 1
HC: Healthy control subjects
hs-CRP: High-sensitivity C-reactive protein
ID2: Inhibitor of DNA binding 2
IL: Interleukin
INA: Indigenous North American
IPA: Ingenuity Pathway Analysis
KLF4: Kruppel-like factor 4
KS: Kolmogorov-Smirnov
miRNA or miR: MicroRNA
mRNA: Messenger RNA
PBMC: Peripheral blood mononuclear cell
PTEN: Phosphatase and tensin homolog
RA: Rheumatoid arthritis
RF: Rheumatoid factor
RISC: RNA-induced silencing complex
TIMP3: Tissue inhibitor of metalloproteinase 3
TP53: Tumor protein 53
UTR: Untranslated region
