# Supplementary material for: Whole blood microRNA expression pattern differentiates patients with rheumatoid arthritis, their seropositive first-degree relatives, and healthy unrelated control subjects
Source: Arthritis Res Ther. 2017 Nov 10;19:249. doi: 10.1186/s13075-017-1459-x (PMC5681796; doi:10.1186/s13075-017-1459-x)
Supplement: Additional file 1: Table S1. — Details of miRNAs included in the study. Table S2. Primers for mRNA targets of miR-103a-3p. Primers were designed using the PrimerQuest tool (Integrated DNA Technologies, Coralville, IA, USA) and the Universal ProbeLibrary system (Roche Life Sciences, Indianapolis, IN, USA) and verified by Primer-BLAST (National Center for Biotechnology Information, Bethesda, MD, USA). 18S ribosomal RNA, F Forward, R Reverse. Table S3. Clinical features of ACPA-positive patients with RA. Table S4. Differentially expressed miRNAs in probands and FDRs: significantly upregulated (↑) and downregulated (↓) or unaltered miRNAs. # Only miRNAs differentially expressed in FDRs compared with patients with RA. Figure S1. IPA target analysis. IPA network showing curated molecular interactions between differentially expressed miRNAs and their experimentally validated target genes. Table S5. Summary of findings derived from IPA regarding gene targets of differentially expressed miRNAs: top molecular and cellular functions, physiological system development and function, diseases and disorders, and networks regulated by significantly modulated miRNAs and their gene targets (P < 0.05 by Fisher’s exact test). Table S6. Spearman’s rank correlation coefficient values and corresponding P values for miR-103a-3p and target mRNAs. ΔCt values of miR-103a-3p Vs target mRNAs from HC, patients with RA, and FDRs were combined for this correlation analysis. Q values represent adjusted P values after applying the Benjamini-Hochberg correction for multiple comparisons. (DOCX 836 kb) [file 13075_2017_1459_MOESM1_ESM.docx]

***Supplementary Data – Contents***

Table S1: Details of miRNAs included in the study

Table S2: Primers for mRNA targets of miR-103a-3p

Table S3: Clinical features of ACPA+ RA patients

Table S4: Differentially expressed miRNAs in probands and FDRs

Figure S1: IPA target analysis

Table S5: Summary of findings from IPA analysis regarding gene targets of differentially expressed miRNAs

Table S6: Spearman correlation co-efficient between miR-103a-3p and target mRNAs

***Table S1: Details of miRNAs included in the study***

| ***miRBase ID*** | ***Assay Name*** | ***Mature miRNA sequence*** | ***Assay ID*** | ***Relevance*** | ***PMID*** |
| --- | --- | --- | --- | --- | --- |
| *hsa-miR-155-5p* | *hsa-miR-155* | *UUAAUGCUAAUCGUGAUAGGGGU* | *002623* | *Up in rheumatoid synovium, PBMCs, synovial fibroblasts; Down in regulatory T -cells in RA* | *27079198, 18383392, 20470394, 23897768, 24151514, 24562503* |
| *hsa-miR-146a-5p* | *hsa-miR-146a* | *UGAGAACUGAAUUCCAUGGGUU* | *000468* | *Deregulated in arthritic serum, PBMCs, macrophages, CD79a+ B-cells, CD3+ T-cells, Th17 cells, synovium* | *27079198, 18383392, 20470394, 23897768, 24562503, 18438844, 20840794* |
| *hsa-miR-26b-3p* | *hsa-miR-26b** | *CCUGUUCUCCAUUACUUGGCUC* | *002444* | *Inhibits synovial fibroblast proliferation and secretion of pro-inflammatory cytokines* | *26088648* |
| *hsa-miR-26b-5p* | *hsa-miR-26b* | *UUCAAGUAAUUCAGGAUAGGU* | *000407* | *Inhibits synovial fibroblast proliferation and secretion of pro-inflammatory cytokines* | *26088648* |
| *hsa-miR-103a-3p* | *hsa-miR-103* | *AGCAGCAUUGUACAGGGCUAUGA* | *000439* | *-n/a-* |  |
| *hsa-miR-323-3p* | *hsa-miR-323-3p* | *CACAUUACACGGUCGACCUCU* | *002227* | *Up in synovial fibroblasts* | *23615628, 22562984* |
| *hsa-miR-19a-3p* | *hsa-miR-19a* | *UGUGCAAAUCUAUGCAAAACUGA* | *000395* | *Down in synovial fibroblasts of RA; regulate TLR2-mediated NF-κB signaling* | *22105995,22684508* |
| *hsa-miR-19b-3p* | *hsa-miR-19b* | *UGUGCAAAUCUAUGCAAAACUGA* | *000396* | *Down in synovial fibroblasts of RA* | *22105995* |
| *hsa-miR-34a-3p* | *hsa-miR-34a** | *CAAUCAGCAAGUAUACUGCCCU* | *002316* | *Down in synovial fibroblasts in RA* | *22161761* |
| *hsa-miR-29a-3p* | *hsa-miR-29a* | *UAGCACCAUCUGAAAUCGGUUA* | *002112* | *Regulate DNA methylation by targeting Dnmt3a and Dnmt3b* | *22334722,23385088* |
| *hsa-miR-29b-3p* | *hsa-miR-29b* | *UAGCACCAUUUGAAAUCAGUGUU* | *000413* | *Regulate DNA methylation by targeting Dnmt3a and Dnmt3b* | *22334722,23385088* |
| *hsa-miR-29c-3p* | *hsa-miR-29c* | *UAGCACCAUUUGAAAUCGGUUA* | *000587* | *Regulate DNA methylation by targeting Dnmt3a and Dnmt3b* | *22334722,23385088* |
| *hsa-miR-152-3p* | *hsa-miR-152* | *UCAGUGCAUGACAGAACUUGG* | *000475* | *Down in rat model of RA; negatively regulates DNMT1* | *22334722,* *25194984* |
| *hsa-miR-203-3p* | *hsa-miR-203* | *GUGAAAUGUUUAGGACCACUAG* | *000507* | *Up in synovial fibroblasts* | *21279994* |
| *hsa-miR-221-3p* | *hsa-miR-221* | *AGCUACAUUGUCUGCUGGGUUUC* | *000524* | *Up in synovial fibroblasts and serum of RA patients* | *22562984*  *25891943* |
| *hsa-miR-222-3p* | *hsa-miR-222* | *AGCUACAUCUGGCUACUGGGU* | *002276* | *Up in synovial fibroblasts* | *22562984* |
| *hsa-miR-223-3p* | *hsa-miR-223* | *UGUCAGUUUGUCAAAUACCCCA* | *002295* | *Up in RA synovium, plasma, T-cells, down in early RA* | *27079198,* *20470394,* *23897768,22903258,19931339* |
| *hsa-miR-18a-5p* | *hsa-miR-18a* | *UAAGGUGCAUCUAGUGCAGAUAG* | *002422* | *Up in RA synovial fibroblasts; involved in cartilage homeostasis* | *23280137* |
| *hsa-miR-24-3p* | *hsa-miR-24* | *UGGCUCAGUUCAGCAGGAACAG* | *000402* | *Up in plasma from RA patients* | *23874885* |
| *hsa-miR-125a-5p* | *hsa-miR-125a-5p* | *UCCCUGAGACCCUUUAACCUGUGA* | *002198* | *Up in plasma and monocytes from RA patients* | *23874885,27014994* |
| *hsa-miR-125a-3p* | *hsa-miR-125a-3p* | *ACAGGUGAGGUUCUUGGGAGCC* | *002199* | *Up in RA synovial monocytes* | *22683424* |
| *hsa-miR-16-5p* | *hsa-miR-16* | *UAGCAGCACGUAAAUAUUGGCG* | *000391* | *Down in RA sera* | *23897768* |
| *hsa-miR-21-5p* | *hsa-miR-21* | *UAGCUUAUCAGACUGAUGUUGA* | *000397* | *Down in T-cells in RA patients; Up in RA synovial fibroblasts* | *25164131,*  *27429986* |
| *hsa-miR-107* | *hsa-miR-107* | *AGCAGCAUUGUACAGGGCUAUCA* | *000443* | *Associated with miR-103a-3p as a cluster* | *20678503* |
| *hsa-let-7e-5p* | *hsa-let-7e* | *UGAGGUAGGAGGUUGUAUAGUU* | *002406* | *Up in synovial monocytes and regulates osteoblast differentiation* | *25601191* |
| *hsa-miR-132-3p* | *hsa-miR-132* | *UAACAGUCUACAGCCAUGGUCG* | *000457* | *Down in plasma of RA patients* | *20470394* |
| *hsa-miR-346* | *hsa-miR-346* | *UGUCUGCCCGCAUGCCUGCCUCU* | *000553* | *Controls release and stability of TNF and IL-18 in synovial fibroblasts* | *21611196,19342689* |
| *hsa-miR-451a* | *mmu-miR-451* | *AAACCGUUACCAUUACUGAGUU* | *001141* | *Down in RA neutrophils; Up in T-cells* | *24574214,24401767* |
| *hsa-miR-15a-5p* | *hsa-miR-15a* | *UAGCAGCACAUAAUGGUUUGUG* | *000389* | *Up in synovial tissues; regulates cell proliferation and apoptosis* | *22100329* |
| *hsa-Let-7a-5p* | *hsa-Let-7a* | *UGAGGUAGUAGGUUGUAUAGUU* | *000377* | *Up in arthritic Th17 cells; down in monocytes* | *18438844,* *26227320* |
| *hsa-miR-26a-5p* | *hsa-miR-26a* | *UUCAAGUAAUCCAGGAUAGGCU* | *000405* | *Up in plasma from RA patients* | *23874885* |
| *hsa-miR-150-5p* | *hsa-miR-150* | *UCUCCCAACCCUUGUACCAGUG* | *000473* | *Up in PBMCs and synovium of RA patients* | *18438844* |
| *hsa-miR-124-5p* | *hsa-miR-124** | *CGUGUUCACAGCGGACCUUGAU* | *002197* | *Down in RA synovial fibroblasts; regulates chemokine secretion and cell proliferation* | *25596157,19404929* |
| *RNU48* | *RNU48* | *GATGACCCCAGGTAACTCTGAGTGTGTCGCTGATGCCATCACCGCAGCGCTCTGACC* | *001006* | *Used as endogenous control for miRNA quantification* | *21407217* |
| *U6 SnRNA* | *U6* | *GTGCTCGCTTCGGCAGCACATATACTAAAATTGGAACGATACAGAGAAGATTAGCATGGCCCCTGCGCAAGGATGACACGCAAATTCGTGAAGCGTTCCATATTTT* | *001973* | *Used as endogenous control for miRNA quantification, regulates spliceosomal assembly* | *23829528,* |
| *RNU44* | *RNU44* | *CCTGGATGATGATAGCAAATGCTGACTGAACATGAAGGTCTTAATTAGCTCTAACTGACT* | *001094* | *Used as endogenous control for miRNA quantification, mapped to intronic regions of genes dysregulated in cancer* | *21407217* |
| *RNU6B* | *RNU6B* | *CGCAAGGATGACACGCAAATTCGTGAAGCGTTCCATATTTTT* | *001093* | *Used as endogenous control for miRNA quantification, mapped to intronic regions of genes dysregulated in cancer* | *21407217* |

**Table S2: Primers for mRNA targets of miR-103a-3p**

| **Gene ID** | **Primer Sequence** | **NCBI Accession ID** | **Amplicon Size (kb)** | **Gene Name** |
| --- | --- | --- | --- | --- |
| *18S* | F 5 -AAAGGAATTGACGGAAGGGCACCA  R 5’-ACCAGACAAATCGCTCCACCAACT | NR_003286.1 | 174 | 18S ribosomal RNA |
| *DICER1* | F 5’ - GTCGTGCCGTATTGGTAGTT  R 5’ - CAGCTCCTCTTGCTCATGTT | NM_030621.4 | 104 | Dicer 1, ribonuclease III |
| *AGO1* | F 5’ - CGGTGTATGCTGAGGTGAAA  R 5’ - AGTCTGAGGTGAGGTCTTGA | NM_012199.4 | 119 | Argonaute 1 |
| *AGO2* | F 5’ - CCTCCGGGAGAACAATCAAA  R 5’ - GGCACTTCTCTGGCTTGATA | NM_012154.3 | 102 | Argonaute 2 |
| *PTEN* | F 5’ - CCCACCACAGCTAGAACTTATC  R 5’ - TCGTCCCTTTCCAGCTTTAC | NM_000314.6 | 109 | Phosphotase and Tensin Homolog |
| *CDK1* | F 5’ – TCACACCGAGTAGTGCATCG  R 5’ - CAAGACTTCGGGTGCTCTGT | NM_001786.4 | 102 | Cyclin dependent kinase 1 |
| *CCNE1* | F 5’ -GATCTCTGTGTCCTGGATGTTG  R 5’ - GCACCACTGATACCCTGAAA | NM_001238.3 | 100 | Cyclin E1 |
| *TIMP3* | F 5’ - GAGTTTGGGTCTTTCTCCTCTG  R 5’ - CTAGAGACCTTGACTGTGCTTG | NM_000362.4 | 104 | Tissue inhibitor of metalloproteinase 3 |
| *CREB1* | F 5’ – GAACCAGCAGAGTGGAGATG  R 5’- GGCATAGATACCTGGGCTAATG | NM_004379.4 | 102 | cAMP responsive element binding protein |
| *TP53* | F 5’ – acacgcttccctggattg  R 5’ - gctcgacgctaggatctgac | NM_000546.5 | 81 | Tumor protein 53 |
| *PANK2* | F 5’ - GGTCTCTGCTGTCTTCTTACTG  R 5’ - GCAGTCCAAACCTCTCATAGTC | NM_153638.3 | 133 | Pantothenate Kinase 2 |
| *PANK3* | F 5’- TACAGGAGGTGGTGCTTACA  R 5’ - AAGCCCTTTACAAGGCAGTC | NM_024594.3 | 99 | Pantothenate Kinase 3 |
| *KLF4* | F 5’ -GACCAGGCACTACCGTAAAC  R 5’ - TTCATGTGTAAGGCGAGGTG | NM_001314052.1 | 99 | Krupppel – like factor 4 |
| *DAPK1* | F 5’ - CGTCATCCTGATCTTGGAACTC  R 5’ - GAAGGGAGTGCAGGTAGTAAAC | NM_004938.3 | 137 | Death associated protein kinase 1 |
| *ID2* | F 5’- GACTGCTACTCCAAGCTCAAG  R 5’ - CAGGTCCAAGATGTAGTCGATG | NM_002166.4 | 105 | Inhibitor of DNA binding 2 |
| *GPD1* | F 5’ – GCTCTGCCAGACTCTCTATCT  R 5’ - CCGGCTAGGAGCATTTAACTC | NM_005276.3 | 106 | Glycerol-3-phosphate dehydrogenase 1 |

Primers were designed using Primer Quest (IDT Technologies) and Universal Probe Library System (Roche Life Sciences) and verified by Primer Blast (NCBI). 18S Ribosomal RNA; F=Forward; R=Reverse

***Table S3: Clinical Features of ACPA+ RA patients***

| ID | Age | Sex | Disease duration (years) | DAS28 scores | CRP  (mg/L) | Anti-CCP | RF  (IU/mL) | Treatment |
| --- | --- | --- | --- | --- | --- | --- | --- | --- |
| RA1 | 56.8 | F | 19.8 | 3.07 | 8.8 | 201 | 289 | Ramipril, Rosuvastatin, Gliclazide, ASA, metformin; Azathioprine, Leflunomide, MTX ((C); HCQ (C) |
| RA2 | 51.8 | F | 12.9 | 1.42 | 1.14 | 201 | 619 | Prednisone (C); Etanercept (C); MTX; Cyclosporin; Leflunomide; SSZ; Vioxx |
| RA3 | 29.8 | F | 20.1 | 1.97 | 7.27 | 201 | 1040 | HCQ (C); Etanercept (C); Leflunomide; SSZ; Gold; Naproxen |
| RA4 | 28.9 | F | 19.5 | 2.24 | 3.47 | 108 | 276 | Enbrel (C); Tylenol, MTX; HCQ; SSZ; Celebrex; Prednisone, Gold |
| RA5 | 39.2 | F | 11.2 | 3.57 | 2.55 | 201 | 569 | SSZ (C); HCQ; Leflunomide; SSZ; Gold; MTX |
| RA6 | 55.4 | F | 0.6 | 4.00 | 4.93 | 201 | 886 | MTX; HCQ (C) |
| RA7 | 54.5 | F | 14.0 | 6.77 | 42.6 | 201 | 287 | Thalidiamine; MTX(C); Prednisone; Arthrotec; HCQ |
| RA8 | 46.5 | F | 0.0 | 4.35 | 2 | 95 | 1540 | MTX (C); HCQ (C); Tylenol #3; SSZ (C); Prednisone; Voltaren; Diclofenac; Vioxx |
| RA9 | 35.9 | F | 5.4 | 4.56 | 27.8 | 201 | 82.8 | Celebrex(C); Tylenol, ASA, Atenolol, Leflunomide, Rosuvastatin, HCQ, Naproxen; MTX; Insulin; SSZ |
| RA10 | 53.1 | F | 14.6 | 4.36 | 4.86 | 201 | 482 | Prednisone(C); Rituximab(C); Etanercept; Gold; AZA; SSZ; Omeprazole (C); Venlafaxine; Diazepam; Amlodipine; Gabapentin; Celebrex; Hydromorphone; Ventolin |
| RA11 | 46.8 | M | 17.7 | 4.68 | 8.73 | 133 | 1010 | MTX (C): Leflunomide; MTX; Naproxen; Tylenol ES |
| RA12 | 46.7 | M | 11.1 | 3.69 | 13.1 | 175 | 141 | HUMIRA (C) HCQ (C); Adalimumab; Apo-Omeprazole; Diclofenac; Flovent HFA; Leflunomide; Salbutamol Sulfate; MTX; Minocycline |
| RA13 | 35.8 | F | 6.0 | 2.90 | 7.44 | 201 | 353 | Active: MTX, HCQ, Tylenol ES |
| RA14 | 70.1 | F | 35.6 | 3.28 | 4.33 | 101 | 1230 | Adalimumab (C); MTX; Vasotec, Omeprazole, Plavix, L-thyroxine, Clonazepam, Fentanyl patch, Percocet; Etanercept, Leflunomide, HCQ, SSZ, Gold |
| RA15 | 29.4 | F | 0.1 | 4.39 | 5.86 | 201 | 148 | Azathioprine, Leflunominde, Orencia, Prednisone, Omeprazole, Tylenol #3, Abatacept (C); Gold, MTX, SSZ, Plaquenil, Prednisone, Enbrel |
| RA16 | 30.5 | M | 2.8 | 5.54 | 6.55 | 76 | 19 | MTX(C); Enbrel (C); prednisone; Tylenol #3; SSZ; HCQ; Leflunomide; Citalopram; Diclofenac; Depomedrol; Naproxen |
| RA17 | 48.2 | F | 6.6 | 3.14 | 16.4 | 201 | 50.5 | Acetaminophen with codeine, Enalapril Maleate, Etanercept, Ferrous Fumarate, Furosemide, Hydrochlorothiazide, HCQ, Metformin, Omeprazole, Teva-Betahistine, |
| RA18 | 32.3 | M | 19.6 | 3.12 | 13 | 19 | 19 | HCQ (C); Naproxen, Plaquenil, SSZ |

*(C) = Current; MTX = methotrexate; HCQ = hydroxychloroquine; SSZ = sulfasalazine*

***Table S4: Differentially expressed miRNAs in Probands and FDRs compared to HCs***

Significantly up-regulated ( ), and down-regulated ( ) or unaltered miRNAs. ^#^ only miRNAs differentially expressed in FDRs compared to RA patients.

***Figure S1: IPA Target Analysis***

***
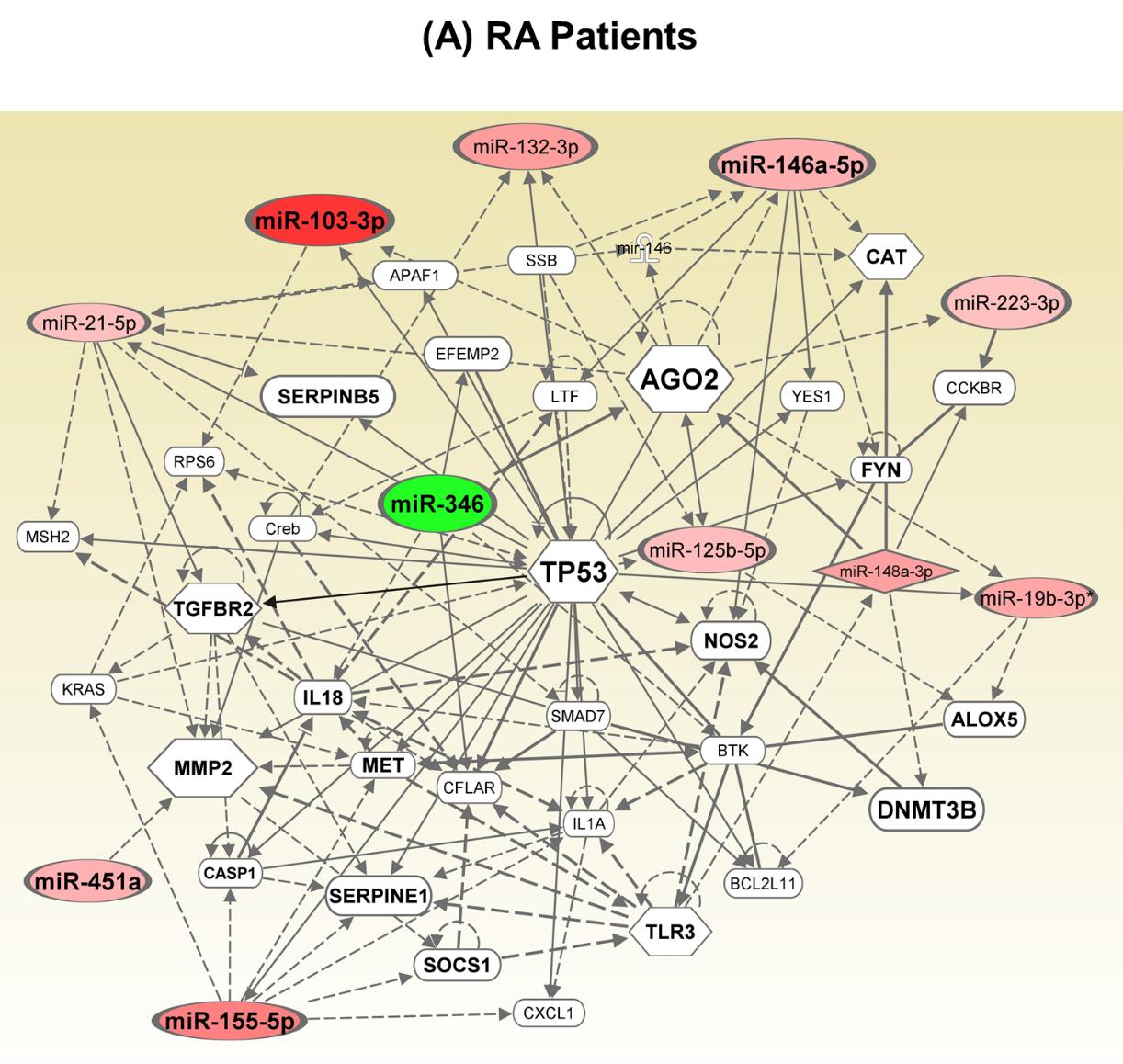
***

IPA analysis network showing curated molecular interactions between differentially expressed miRNAs and their experimentally-validated target genes.

***Table S5: Summary of findings from IPA analysis regarding gene targets of differentially expressed miRNAs***

Table summarizing top molecular and cellular functions, physiological system development and function, diseases and disorders and networks regulated by significantly modulated miRNAs and their gene targets (*P*<0.05; fisher’s exact test).

***Table S6: Spearman correlation coefficients between miR-103a-3p and target mRNAs***

Table showing Spearman rank correlation coefficient values and corresponding *P*-values miR-103a-3p and target mRNAs. ΔC_t_ values of miR-103a-3p Vs target mRNAs from HC, RA patients and FDRs were combined for this correlation analyses. *Q*-values represent adjusted *P*-values after applying Benjamini-Hochberg correction for multiple comparison.
